# Leukonychia and peeling skin in an 11-year-old girl

**DOI:** 10.1016/j.jdcr.2026.03.028

**Published:** 2026-04-09

**Authors:** Ziad Alharbi, Sahar Alshomer, Faisal Alghamdi, Aljohara Alkhenaizan, Mohammed AlBalwi, Rahaf Bashihab, Sultan Al-Khenaizan

**Affiliations:** a King Saud bin Abdulaziz University for Health Sciences, College of Medicine, Riyadh, Saudi Arabia; b Department of Dermatology, King Abdulaziz Medical City, Ministry of National Guard Health Affairs, Riyadh, Saudi Arabia; c College of Medicine, Imam Mohammad Ibn Saud Islamic University (IMSIU), Riyadh, Saudi Arabia; d Department of Pathology and Laboratory Medicine, King Abdulaziz Medical City, National Guard Health Affairs, Riyadh, Saudi Arabia; e King Abdullah International Medical Research Center, Medical Genomic Research Department, Riyadh, Saudi Arabia; f Department of Pediatrics, King Abdulaziz Medical City, Ministry of National Guard Health Affairs, Riyadh, Saudi Arabia

**Keywords:** acral keratoses, CAST gene, hypotrichosis, leukonychia, pediatric dermatology, PLACK syndrome

## Case description

**Fig 1:**
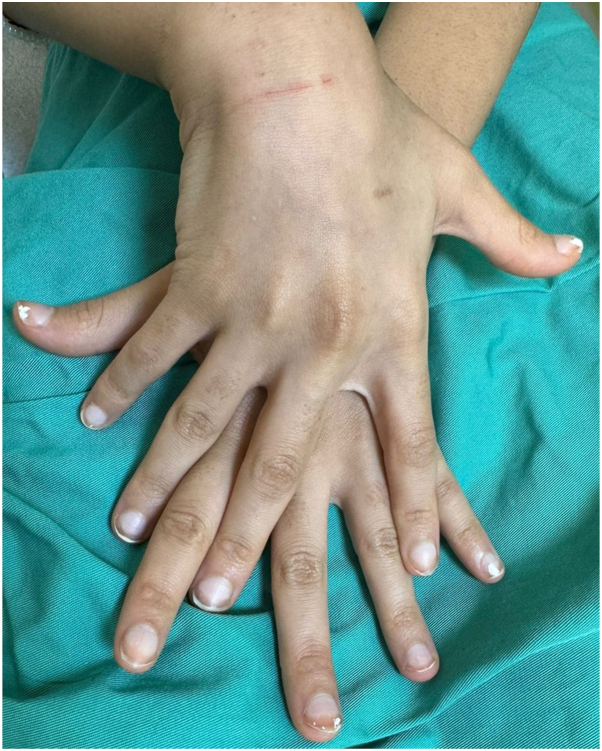
Fingernails showing leukonychia.

**Fig 2:**
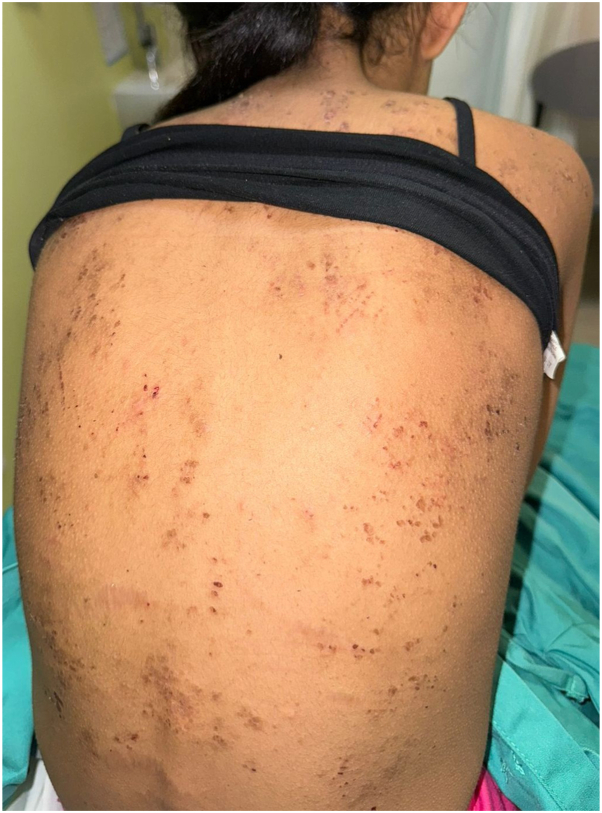
Skin examination revealed generalized xerosis with follicular hyperkeratosis.

**Fig 3:**
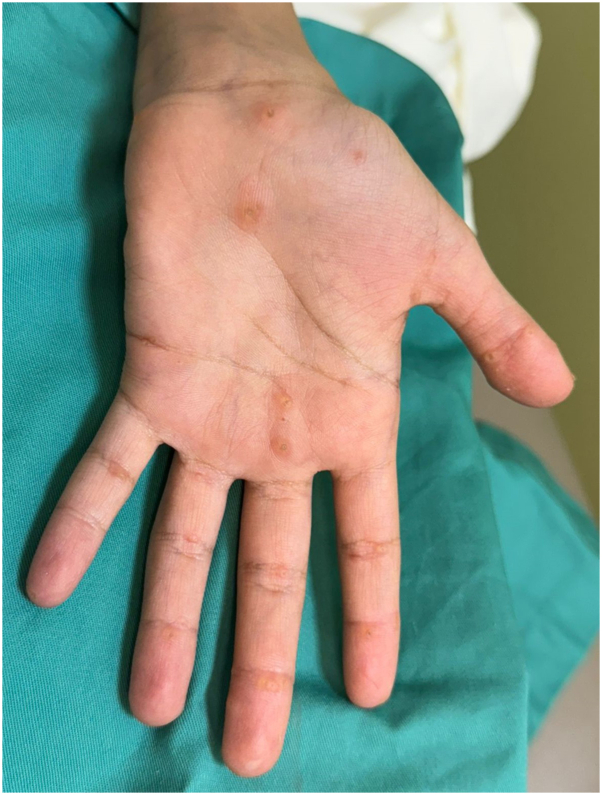
Hyperkeratotic papules on palms.

A previously healthy 11-year-old Saudi girl presented to another hospital with progressive lethargy, abdominal pain, vomiting, jaundice, and leg swelling. Echocardiography revealed a severely dilatated cardiomyopathy (DCM) with severely depressed left ventricular ejection fraction of 10%, for which she was transferred to our institution, King Abdullah Specialized Children’s Hospital, where she underwent orthotopic heart transplantation. Family history was significant for first-degree consanguinity and the sudden death of a sister at the age of 2 years. A dermatologic consultation was requested to evaluate skin findings. Skin examination revealed generalized xerosis with follicular hyperkeratosis, cheilitis, and multiple hyperkeratotic papules on palms and soles. Fingernails and toenails showed leukonychia with unremarkable hair examination.(Fig 1, Fig 2, Fig 3)

**Question 1: Which of the following is the most likely diagnosis?**

**A.** Carvajal syndrome
**B.** Naxos syndrome
**C.** PLACK syndrome
**D.** Vitamin A deficiency
**E.** Zinc deficiency

**Answer:**

Correct answer: **C**

## Discussion

PLACK syndrome is an acronym for peeling skin, leukonychia, acral punctate keratoses, cheilitis, and knuckle pads.^1^ It is an autosomal recessive genetic disease caused by mutations in the calpastatin (CAST) gene.^1^ Our patient's skin findings were highly suggestive of PLACK syndrome, which was confirmed by identifying a homozygous c.1054dup (p.Arg352ProIlaTer4) mutation in the CAST gene. Naxos and Carvajal syndromes are characterized by woolly hair, palmoplantar keratoderma, and right arrhythmogenic cardiomyopathy or left dilated cardiomyopathy, respectively. Woolly hair was not seen in our patient. Leukonychia is not a feature of vitamin A nor zinc deficiencies.

Echocardiography in our patient revealed a severe DCM with severely depressed left ventricular ejection fraction, for which she underwent orthotopic heart transplantation. Recently, there have been at least 2 reports describing DCM in PLACK syndrome patients,^2^^,^^3^ with 1 report describing heart transplantation as in our patient.^3^ This report adds emphasis to a potential link between PLACK syndrome and cardiomyopathy. Calpastatin is the endogenous inhibitor of calpains, calcium-dependent proteases involved in cytoskeletal remodeling and keratinocyte integrity. Loss of calpastatin leads to unopposed calpain activity and excessive proteolysis of structural proteins, resulting in impaired epidermal barrier stability and the characteristic skin findings of PLACK syndrome.^4^ Calpains are known to play a critical role in the dynamic remodeling of the cardiac myocyte cytoskeleton. Excessive calpain activity may also lead to the degradation of key cytoskeletal proteins such as desmin and titin in myocardial fibers, leading to myocardial fibrosis and impaired contractility.^4^ In our patient, the explanted native heart histopathology revealed extensive ventricular and atrial fibrosis.

To date, there are 27 patients with PLACK syndrome reported, including ours, with 7 patients developed DCM. Among these, 3 had heart transplantation, 1 required a biventricular assist device, and 3 succumbed to progressive heart failure. The reported age of onset of DCM ranged from 4 to 15 years. Investigating patients with PLACK syndrome for DCM is probably warranted, as it seems more than a coincidence, but further research is needed.

Management of PLACK syndrome remains symptomatic and supportive, primarily with emollients and keratolytics. Sawan et al^5^ described a child with PLACK syndrome, who was treated with intravenous lipid emulsion (Vitalipid plus Lipofundin-MCT/LCT 20%), resulting in dramatic improvement in skin manifestations. They postulated that intralipid therapy may help in stabilizing keratinocyte membranes and partially reverse cellular dysfunction in PLACK syndrome patients’ fibroblasts.^5^ Whether intralipid treatment can help cardiomyopathy in PLACK syndrome remains open for investigation.

## Conflicts of interest

None disclosed.
